# Evaluating healthcare professionals’ perceptions of dispensing separation and sale of pharmacy medicine in Brunei Darussalam

**DOI:** 10.1186/s40545-023-00594-5

**Published:** 2023-08-24

**Authors:** Rabi’atul Nur Amalia Abdullah Juperi, Hui Poh Goh, Inayat Ur Rehman, Kah Seng Lee, Long Chiau Ming, Andi Hermansyah

**Affiliations:** 1 PAP Rashidah Sa’adatul Bolkiah Institute of Health Sciences, Universiti Brunei Darussalam, Gadong, BE1410 Brunei; 2 Department of Pharmacy, Abdul Wali Khan University Mardan, Mardan, 23200 Pakistan; 3 Faculty of Pharmacy, University of Cyberjaya, Cyberjaya, Malaysia; 4 Department of Pharmacy Practice, Faculty of Pharmacy, Universitas Airlangga, Surabaya, 60115 Indonesia; 5 School of Medical and Life Sciences, Sunway University, Bandar Sunway, 47500 Petaling Jaya, Selangor Malaysia

**Keywords:** Perception, Awareness, Pharmacy (P) medicine, Medicine access, Dispensing separation

## Abstract

**Background:**

Pharmacy medicine (P) is obtained exclusively from a pharmacy under the supervision of a pharmacist. This study aims to understand the perception of healthcare professionals towards the dispensing separation, as well as the dispensing of pharmacy medicine by community pharmacies to enhance patient health outcomes in Brunei Darussalam.

**Methods:**

A cross-sectional study was conducted between 1st March 2023 and 20th April 2023 among healthcare professionals. A newly designed and validated questionnaire was used. Its face and content validity, along with internal consistency, was adequately established. Convenient sampling was employed to recruit participants for the study. Statistical analysis using one-way ANOVA was performed, considering a *p*-value < 0.05 as statistically significant.

**Results:**

The study compiled data from 108 participants, comprising doctors (38.9%) and pharmacy technicians (45.4%). Approximately 28.7% of respondents had 11–20 years of healthcare experience, while 25.9% had less than 5 years. Nearly all respondents (98.1%) agreed on the vital role pharmacists and pharmacy technicians play in prescription checks. A significant number of participants (93.5%) agreed that Brunei’s current medicine dispensing system needs improvement. The mean total score for the perception of medicine dispensing in Brunei was 3.79 ± 1.103. A statistically significant difference was found between the perception score and the respondents’ profession (*p* = 0.018), but not with their age, experience, or place of work. Respondents’ awareness score showed no statistically significant correlation with their profession, age, experience, or place of work.

**Conclusion:**

The study underscores the necessity for more patient-centered care in community pharmacies in Brunei Darussalam. The country’s healthcare professionals should recognize the potential advantages of expanding pharmacy services. However, to implement these services successfully, regulatory restrictions and infrastructure limitations must be addressed.

## Background

Pharmacy services an integral to the health care system [1]. Traditionally, pharmacists were primarily responsible for the manufacture and supply of medicines [2]. However, due to globalization and advancements in healthcare, the role of pharmacists has evolved towards clinically oriented services [2, 3]. Due to shift in the responsibilities and role of pharmacist, the community pharmacists are also playing their active role in dispensing, counselling on correct use, storage of medicines and retailing of medicines [3–6]. Despite professional training, the capabilities of community pharmacists are often under-utilized [7]. A cross-country comparison (Australia, Canada, England, the Netherlands, Scotland, and the United States) shows that community pharmacists can improve health by promoting medication adherence, reducing drug-related adverse events, and reducing provider visits, hospitalizations, and readmissions [7].

Community pharmacy is the most accessible and widely distributed of the various health care facilities [8]. Although community pharmacists have a rich tradition of providing health-related services, advancements in knowledge and technology have raised expectations for delivering expanded primary health services [9]. Community pharmacists are involved in a wide variety of professional activities that optimizes medication therapy and promotes health and disease prevention [8]. Community pharmacists successfully embraced and implemented the practices of expended clinical services, as evident from findings of a systematic review [10]. The dispensing process in community pharmacy is a major part of the quality use of medicines and the provision of medicine information in the form of verbal or written information [11]. The changes in the structural and organizational of the community pharmacy services require the incorporation of a new practice into the routine workflow [12]. A consultation room and additional experienced staff may be required to attend to the clinical activities [9].

Community pharmacist has the potential to assist the patients in management of chronic diseases [13], and the community pharmacy acts as an optimal environment for the pharmacists to fulfill their professional responsibilities [14]. Community pharmacies have the potential to act as convenient and easily accessible sites for patient-centered medication management services [15]. According to the Ministry of Health Brunei Darussalam, the community pharmacies in Brunei are limited to dispensing medications as prescribed by doctors in accordance with the Good Dispensing Practice, providing patient counselling about their medications, and supplying floor stock medications to peripheral clinics [16].

The separation of prescribing and dispensing is a contentious topic, as prescribing involves physicians, while dispensing forms an integral part of services rendered by pharmacists [17]. These two processes—prescribing and dispensing of medication—while vital, represent distinct phases in the medication management cycle [18]. The dispensing entails several crucial tasks, including prescription verification, screening for drug interactions, resolution of drug-related problems, documentation of patient medication records, counseling on proper medication use, and drug therapy monitoring [19]. In Brunei Darussalam the health facilities and services are provided by the government [20]. Although literature offers no specific data on the separation of prescribing and dispensing in Brunei, prior studies have documented a gradual shift towards this separation in neighboring countries such as Malaysia, Japan, Taiwan, Indonesia, Korea, and the Philippines [21–24]. Dispensing separation is believed to contribute to the reduction of medical costs, population health improvement, and enhanced healthcare quality [17].

In Brunei Darussalam, the medicinal products manufactured, sold, supplied, or imported into the country are regulated under the Medicines Order, 2007 and other related regulations. Medicines are classified into three categories, namely Prescription medicines (POM), Pharmacy medicine (P), and General Sales List (GSL) [25]. POMs are medicines that can only be prescribed by doctors and dispensed upon presentation of a complete and valid prescription. P medicines, on the other hand, are available exclusively from pharmacies and must be purchased in the presence of a pharmacist. Typically, these are stored behind the counter in pharmacies and not in open areas. GSL medicines can be sold in various outlets, such as retail stores, supermarkets, or any other shops [26]. Given the lack of data and evidence on healthcare professionals’ perception of prescription and dispensing of pharmacy medicine at community pharmacies, there is a compelling need for this study. It aims to gauge perceptions on the separation of prescribing and dispensing, as well as the dispensing of pharmacy (P) medicine by community pharmacies, to enhance patient health outcomes in Brunei Darussalam.

## Methods

### Study design

A cross-sectional study was conducted from 1st March 2023 until 20th April 2023 among healthcare professionals, including medical doctors, dentists, pharmacists, and pharmacy technicians, from government health centers across four districts in Brunei Darussalam.

### Study population

This study employed a convenience sampling strategy. In this non-probability sampling approach, inclusion in the study was primarily based on the convenience and willingness of potential participants. This method does not require a predetermined sample size and does not aim to be fully representative of the population. The goal was to expedite data collection and gain insights from an easily accessible group of individuals possessing characteristics relevant to the research question.

### Study instrument and data collection

Due to the lack of a validated questionnaire aligning with our study objective, we developed one through literature review and a Delphi technique application. This expert panel comprised healthcare professionals who provided input and feedback on the questionnaire’s content, readability, and comprehension by the participants. After satisfying the Delphi technique steps, the questionnaire was tested for content and face validity among a limited population. Cronbach’s alpha was used to evaluate the questionnaire’s internal consistency. The final questionnaire consisted of five sections with a total of 19 questions: (1) five questions on sociodemographic data (profession, age, duration of practice, place of work, and country where participants completed their professional training); (2) five questions on perceptions of medicine dispensing in private practice; (3) three questions on perceptions of Brunei’s healthcare system; (4) three questions on awareness and perceptions towards the separation of prescribing and dispensing; and (5) three questions about the perception of Pharmacy (P) medicine. The questions, primarily closed-ended, offered two choices: ‘Yes’ and ‘No’. For each section, ‘Yes’ and ‘Aware’ were scored as one point, while ‘No’ and ‘Unaware’ were scored as zero. The overall perception score is the cumulative score for sections two, three, and five, with scores ranging from 0 to 13. A higher score indicates a better Perception score.

The questionnaires were distributed both online (via social media platforms like Facebook, WhatsApp) and in-person at the selected hospitals. Participants provided informed consent, and the study’s objectives were explained to them prior to signing the consent.

### Pilot testing

Face and content validation were conducted among a small targeted sample of randomly selected respondents who met the inclusion criteria. All participants found the questionnaire items to be simple, clear, and aligned with the objectives. Content validity was assessed to determine the degree to which the instrument covers all items necessary to reflect the construct of interest. Each item was reviewed by the aforementioned experts who rated the content validity of each item in terms of its relevance. The internal consistency of the questionnaire was assessed by a Cronbach’s alpha value of 0.762, indicating adequate reliability.

### Data analysis

Data analysis was performed using SPSS version 21. Descriptive statistics summarized the data into percentages, frequencies, means, and standard deviations. One-way ANOVA tests were utilized for comparisons among different demographic groups, with a *p*-value < 0.05 considered statistically significant.

### Ethical approval

This study received full approval from the Joint Research Ethics Committee of the PAPRSB Institute of Health Sciences, Universiti Brunei Darussalam (UBD/PAPARSBIHSREC/2022/115). Additionally, data collection for this study was executed in an anonymous and confidential manner.

## Results

### Demographic data

The study collected data from a total of 108 participants, with their demographic details tabulated in Table 1. The majority of the participants were pharmacy technicians (45.4%), followed by doctors (38.9%). The largest age group represented was 20–30 years (33.3%). A significant proportion of the participants (28.7%) had 11–20 years of professional experience (Table 1). The majority of participants received their education in Brunei Darussalam (35.2%), followed by the United Kingdom (13.9%). About half of the participants worked at Suri Seri Begawan (SSB) Hospital, followed by 24.1% in government health centers (details in Table 1).

**Table 1:** Sociodemographic details of participants (*n* = 108)

Variable	*N* (%)
Profession	
Doctor	42 (38.9)
Dentist	3 (2.8)
Pharmacist	14 (13.0)
Pharmacy technician	49 (45.4)
Age in years	
20–30	36 (33.3)
31–40	30 (27.8)
41–50	21 (19.4)
51–60	11 (10.2)
> 60	10 (9.3)
Experience in years	
0–4	28 (25.9)
5–10	21 (19.4)
11–20	31 (28.7)
21–30	14 (13)
30–40	10 (9.3)
> 40	4 (3.7)
Country of study	
Australia	2 (1.9)
Bangladesh	3 (2.8)
Brunei Darussalam	38 (35.2)
Egypt	2 (1.9)
India	11 (10.2)
Malaysia	2 (1.9)
Myanmar	9 (8.3)
New Zealand	3 (2.8)
Pakistan	8 (7.4)
Philippines	5 (4.7)
Singapore	10 (9.3)
United Kingdom	15 (13.9)
Place of work	
RIPAS	16 (14.8)
PMMPMHAMPB	7 (6.5)
SSB	53 (49.1)
PIHM	1 (0.9)
Government health centers	26 (24.1)
DPS	5 (4.6)

### Perception of the medicine dispensing in Brunei

With respect to perceptions of medicine dispensing in Brunei, almost all the participants (98.1%) affirmed the crucial role of pharmacists and pharmacy technicians in checking prescriptions. A considerable majority of participants (93.5%) agreed that the current medicine dispensing system in Brunei needs improvement. Additionally, 80.6% of participants expressed that more medicines should be sold as over-the-counter (OTC) options at stores. A significant proportion of participants (53.7%) believed that more medicines should not be sold without a prescription from a pharmacist. Nevertheless, 60.2% agreed that certain medicines, such as antihistamines, medicated cough syrup, antibiotic cream, or eye drops, should be available without a prescription at community pharmacies (details in Table 2). The overall total score for the perception of medicine dispensing in Brunei was 3.79 ± 1.103 (mean ± SD).

**Table 2:** Responses to the perception of medicine dispensing (*n* = 108)

Question	No (*n*)	%	Yes (*n*)	%
6. Do you agree that pharmacists have an important role in checking prescriptions?	2	1.9	106	98.1
7. Do you feel the current medicine dispensing system in Brunei should be improved?	7	6.5	101	93.5
8. Do you think that more medicine should be sold as over-the-counter medicine at the shop/store?	21	19.4	87	80.6
9. Do you think that prescription-only medicine should be sold without a prescription by a pharmacist?	58	53.7	50	46.3
10. Do you think that more medicine such as antihistamines, medicated cough syrup, antibiotic cream, or eye drops should be supplied without a prescription by a community pharmacy?	43	39.8	65	60.2
Total score (mean ± SD)	3.79 ± 1.103			

### Perception of Brunei’s healthcare system

In terms of respondents’ perception of Brunei’s healthcare system, a majority agreed that the medicine supply system was effective in both public (73.1%) and private (72.2%) healthcare institutions. When asked about their use of private general practitioners (GPs) or private pharmacies, 41.7% reported no use, while more than half of the respondents (54.6%) reported usage less than five times per year (details in Table 3). The overall score for the perception of Brunei’s healthcare system was 2.08 ± 0.908 (mean ± SD).

**Table 3:** Responses to the perception of Brunei’s healthcare system (*n* = 108)

Question	No (*n*)	%	Yes (*n*)	%
11. Do you feel the medicine supply system in public healthcare institutions/hospitals is effective?	29	26.9	79	73.1
12. Do you feel the medicine supply system in private healthcare/hospitals is effective?	30	27.8	78	72.2
13. How often do you use private GP/private pharmacies				
None	45	41.7		
Less than 5 times per year	59	54.6		
Less than 5 times per month	3	2.8		
Less than 5 times per week	1	0.9		
Total score (mean ± SD)	2.08 ± 0.908			

### Awareness and perception towards the separation of prescribing and dispensing

Regarding awareness and perception towards the separation of prescribing and dispensing, 73.1% of the respondents indicated their awareness of the practice in both developed countries and Brunei’s government sector. Furthermore, 80.6% agreed that full separation of prescribing and dispensing should be implemented in Brunei for the private sector (details in Table 4). The mean total score for the perception of the separation of prescribing and dispensing was 2.27 ± 0.871 (mean ± SD).

**Table 4:** Responses to the awareness and perception towards the separation of prescribing and dispensing (*n* = 108)

Question	Aware (*n*)	%	Unaware (*n*)	%
14. Are you aware of the separation of prescribing and dispensing in developed countries?	79	73.1	29	26.9
15. Are you aware of the separation of prescribing and dispensing in Brunei’s public sector?	79	73.1	29	26.9
16. Do you agree that full separation of prescribing and dispensing should be implemented in Brunei (for private sectors)	87	80.6	21	19.4
Total score (mean ± SD)	2.27 ± 0.871			

### Perception towards P-medicine

Upon evaluation of respondents’ perception towards P Medicine, it was observed that 84.3% were aware of the different product classifications: (1) Pharmacy Medicine (P Medicine), (2) Prescription-Only Medicine (POM), and (3) General Sales List (GSL). Additionally, 77.8% were aware of the distinctions between each of these classifications. Notably, 87.0% of participants expressed the desire to increase the number of P Medicines available in the country to improve access to medicines (details in Table 5). The mean total score for the perception towards P Medicine was 2.49 ± 0.755 (mean ± SD).

**Table 5:** Responses to the perception towards P-medicine (*n* = 108)

Question	Aware/yes (*n*)	%	Unaware/no (*n*)	%
17. Are you aware of the different product classifications?	91	84.3	17	15.7
18. Are you aware of the differences between the three different classifications?	84	77.8	24	22.2
19. Do you think that MOH should increase the number of P-medicine available in Brunei to achieve medicine access?	94	87.0	14	13.0
Total score (mean ± SD)	2.49 ± 0.755			

The comparison of the overall mean perception score with different demographic groups (profession, age, experience in years, place of work) revealed a statistically significant difference according to profession (*p* = 0.018). Pharmacists achieved a higher score (mean ± SD) of 9.43 ± 1.505, followed by dentists with 8.67 ± 0.577 (as shown in Table 6). However, when comparing the overall mean awareness score with different demographic groups (profession, age, experience in years, place of work), no statistically significant difference was observed in respondents’ awareness scores with regard to their profession, age, experience, or place of work (as shown in Table 7).

**Table 6:** Comparison of the overall mean perception score with different demographic groups (profession, age in years, experience in years, place of work)

Comparison of perception score	Mean ± SD	*P*-value
Profession		
Pharmacist	9.43 ± 1.505	0.018*
Pharmacy technician	8.51 ± 1.583	
Doctor	7.81 ± 1.550	
Dentist	8.67 ± 0.577	
Age in years		
Less than 30 years	8.58 ± 1.628	0.28
31–40 years	8.40 ± 1.653	
41–50 years	8.62 ± 1.322	
51–60 years	7.55 ± 1.864	
More than 60 years	7.80 ± 1.169	
Experience in years		
Less than 5 years	8.45 ± 1.594	0.512
5–10 years	8.70 ± 1.593	
11–15 years	8.65 ± 1.461	
16–20 years	8.00 ± 1.809	
21–25 years	8.00 ± 2.000	
More than 25 years	7.95 ± 1.605	
Place of work		
Government health clinic	8.28 ± 1.709	0.972
RIPAS hospital	8.88 ± 1.628	
SSB hospital	8.11 ± 1.589	
PMMPMHAMPB	8.86 ± 1.069	
DPS	9.67 ± 1.528	

Tested using one-way ANOVA (analysis of variance) * statistically significant (*p* < 0.05)

**Table 7:** Comparison of the overall mean awareness score with different demographic groups (profession, age in years, experience in years, place of work)

Comparison of awareness score	Mean ± SD	*P*-value
Profession		0.585
Pharmacist	2.43 ± 0.756	
Pharmacy technician	2.33 ± 0.801	
Doctor	2.17 ± 1.101	
Dentist	–	
Age in years		0.216
Less than 30 years	2.44 ± 0.652	
31–40 years	2.23 ± 0.935	
41–50 years	2.29 ± 0.902	
51–60 years	2.18 ± 1.168	
More than 60 years	1.80 ± 0.919	
Experience in years		0.171
Less than 5 years	2.34 ± 0.670	
5–10 years	2.50 ± 0.761	
11–15 years	2.25 ± 0.910	
16–20 years	2.08 ± 1.084	
21–25 years	2.86 ± 0.378	
More than 25 years	1.85 ± 1.040	
Place of work		0.88
Government health clinic	2.28 ± 0.922	
RIPAS hospital	2.13 ± 0.957	
SSB hospital	2.30 ± 0.822	
PMMPMHAMPB	2.29 ± 0.951	
DPS	2.33 ± 1.155	

Tested using one-way ANOVA (analysis of variance)

## Discussion

Our study sought to assess healthcare professionals’ perception of the demand for community pharmacy dispensing by increasing the availability of pharmacy medicine (P) in Brunei Darussalam. The shift in the pharmacist’s role from traditional towards patient-centric care services and community pharmacy imposes additional responsibilities. These include ensuring the dispensing of rational therapy to reduce the disease burden, minimizing chances of error, providing adequate counseling on dispensed pharmacy medicines, discouraging the dispensing of prescription-only medicines (POM) without prescriptions, and ensuring effective dispensing and counseling on over-the-counter (OTC) medicines to the community. Overall, community pharmacists act as healthcare team members safeguarding public health and assisting physicians and other healthcare members in ensuring the dispensing of rational therapy for optimum therapeutic outcomes.

Our findings revealed that a majority (80.6%) of the participants were of the opinion that more medicines should be sold as over-the-counter medicines at shops/stores. However, another study reported an increase in the misuse of over-the-counter medicines, which is of concern due to patient safety issues [27]. Community pharmacists are critical to the provision of education on safe and effective use of over-the-counter medicine [28]. Our study also found that the majority of participants believed that more medicines such as antihistamines, medicated cough syrup, antibiotic cream, or eye drops should be supplied without a prescription by a community pharmacy. However, previous literature indicated that in Brunei, as in other Asian countries, OTC medicines can be directly purchased off the shelf in stores [29].

Proper understanding and knowledge about over-the-counter medicines are vital for appropriate outcomes. A study in Brunei Darussalam reported that while the majority of university students demonstrated sound knowledge and attitude towards taking over-the-counter medicines, gaps in appropriate practices still existed, including not reading medicine labels properly, checking for expiry dates, and self-medication [29]. According to the Ministry of Health in Brunei Darussalam, community pharmacies are limited to dispensing medications as prescribed by doctors in accordance with Good Dispensing Practice, providing patient counseling about their medications, and supplying floor stock medications to peripheral clinics [16].

In Brunei Darussalam, there is a lack of literature documenting the practice of prescribing and dispensing separation, making it challenging to compare our findings with other local studies due to limited data availability. However, other countries like Malaysia, Japan, Taiwan, Indonesia, Korea, and the Philippines have gradually begun to adopt prescribing and dispensing separation practices [21–24]. This study disclosed that a majority of participants (73.1%) are aware of the dispensing separation practiced in both developed countries and Brunei’s government sector. Furthermore, 80.6% of participants concurred that full separation of prescribing and dispensing should be implemented in Brunei’s private sectors. According to a study done in Malaysia, approximately more than half of the respondents were also aware that the separation of prescribing and dispensing occurs in other developed countries (67.5%) and the Malaysian public sector (70.9%) [21]. Our findings, in which 80.6% of participants agreed that full separation of prescribing and dispensing should be implemented in Brunei’s private sectors, are consistent with these results [21]. However, due to a dearth of specific studies on dispensing separation and pharmacy medicine in Brunei Darussalam and other countries, it is challenging for us to compare our findings with similar studies.

### Limitations and strength of study

Notably, the lack of documentation concerning prescribing and dispensing separation in Brunei makes it difficult to compare our findings with other local studies. Nevertheless, the majority of our study participants were aware of dispensing separation practices, mirroring trends in developed countries and other nations like Malaysia, Japan, Taiwan, Indonesia, Korea, and the Philippines. A significant portion of respondents also advocated for the full implementation of prescribing and dispensing separation in Brunei’s private sectors, similar to the attitudes observed in studies from other countries.

The present study has some limitations, including a relatively small sample size, lack of gender data, and an uneven distribution of respondents among different healthcare professions. We recommend that future research employ larger, more diverse sample sizes for more comprehensive information on dispensing separation and community pharmacy practices.

However, the strength of this study lies in its novelty—it is the first of its kind in Brunei Darussalam, providing valuable insights to the Ministry of Health and policy makers. These findings should be taken into account when formulating future policies for the betterment of public health in Brunei.

Further research is warranted to uncover the most effective strategies for implementing expanded community services in Brunei, taking into account the unique challenges and opportunities inherent in the local healthcare system. By addressing these issues, community pharmacists in Brunei may play a more significant role in improving patient outcomes, promoting medication adherence, and ultimately, enhancing the overall quality of healthcare in the country.

## Conclusion

In conclusion, this study underlines the need to transition towards a more patient-centered care model in Brunei’s community pharmacies. There is an imperative requirement for healthcare professionals in the country to acknowledge the potential advantages of broadening pharmacy services. Furthermore, they should be proactive in enhancing the roles and responsibilities of community pharmacists. However, to successfully implement these extended services, it is crucial to address various obstacles and challenges, such as regulatory constraints and infrastructural limitations.

## Data Availability

Please contact author for data requests.

## Abbreviations

DS: Dispensing separation
POM: Prescription-only medicines
P-medicine: Pharmacy medicine
GSL: General Sales List
GP: General practitioners
UBD: Universiti Brunei Darussalam
PAPRSB IHSREC: Brunei; Pengiran Anak Puteri Rashidah Institute of Health Sciences Research Ethics Committee
RIPAS: Raja Isteri Pengiran Anak Saleha (RIPAS)
PMMPMHAMB: Pengiran Muda Mahkota Pengiran Muda Haji Al-Muhtadee Billah
PIHM: Pengiran Isteri Hajjah Mariam
DPS: Department of Pharmaceutical Services
